# Adherence to UK dietary guidelines in school-aged children from the Avon Longitudinal Study of Parents and Children (ALSPAC) cohort

**DOI:** 10.1017/S0007114522003336

**Published:** 2023-08-14

**Authors:** Genevieve Buckland, Kate Northstone, Pauline M. Emmett, Caroline M. Taylor

**Affiliations:** 1 Centre for Academic Child Health, Bristol Medical School, University of Bristol, Bristol, UK; 2 Department of Population Health Sciences, Bristol Medical School, University of Bristol, Bristol, UK

**Keywords:** Avon Longitudinal Study of Parents and Children (ALSPAC), UK Eatwell guide, Dietary guidelines, School-aged children, Socio-demographic predictors, Inequality

## Abstract

Compliance to UK dietary recommendations was assessed in school-aged children from a population-based cohort: the Avon Longitudinal Study of Parents and Children (ALSPAC). A Children’s Eatwell Guide (C-EWG) score was developed to assess socio-demographic predictors of meeting dietary recommendations. ALSPAC children with plausible diet diary data at 7 years (*n* 5373), 10 years (*n* 4450) and 13 years (*n* 2223) were included in the study. Their dietary intakes (recorded between 1998 and 2006) were compared with dietary guidelines for total and saturated fats, free sugars, salt, fibre, protein, carbohydrates, fruit and vegetables, non-oily and oily fish and red/processed meat. The C-EWG score (0–9 points) indicated the number of recommendations met at each age. Cross-sectional associations between socio-demographic characteristics and C-EWG scores were assessed using multivariable regression. The lowest adherence to guidelines at 7 years was for sugar (0·1 % meeting recommendations), followed by fibre (7·7 %), oily fish (9·5 %), saturated fat (9·7 %) and fruit and vegetables (15·2 %). Highest adherence was for limiting red/processed meat (67·3 %) and meeting carbohydrate recommendations (77·3 %). At 7 years, 12·1 % of participants failed to meet any of the nine recommendations, 26·9 % met one and 28·2 % met two. Similar patterns were seen at 10 and 13 years. A lower social class and maternal educational attainment and higher maternal BMI were associated with meeting fewer recommendations. Most school-aged children in this cohort did not meet UK dietary recommendations, particularly children from lower socio-economic backgrounds. Additional public health initiatives are needed to improve the quality of UK children’s diets, particularly targeting lower socio-economic groups.

National dietary recommendations aim to encourage dietary intakes that will improve the well-being and long-term health of their population. The UK dietary guidelines are developed by the UK Health Security Agency in partnership with the Scientific Advisory Committee on Nutrition and the predecessor group, The Committee on Medical Aspects of Nutrition Policy^(1)^. Their current recommendations for adults^(1–3)^ align closely to international dietary recommendations^(4)^ and are detailed in Table 1. These dietary guidelines are visually represented within the UK’s Eatwell Guide (EWG), launched in 2016, to help inform the population on how to meet dietary recommendations^(2)^.

**Table 1.** UK dietary recommendations for key nutrients and foods within the Eatwell Guide, including age-adjusted portion sizes calculated for children at 7-, 10- and 13 years of age within the Avon Longitudinal Study of Parents and Children (ALSPAC) study

UK Dietary Recommendations	Age-specific recommendations and age-appropriate portion sizes		
	7-year-olds	10-year-olds	13-year-olds
Nutrients			
Total fat*	≤ 35 % total energy	≤ 35 % total energy	≤ 35 % total energy
Saturated fat*,†	≤ 11 % food energy	≤ 11 % food energy	≤ 11 % food energy
Free sugars‡	≤ 5 % total energy	≤ 5 % total energy	≤ 5 % total energy
Salt§	≤ 5 g/d	≤ 5 g/d	≤ 6 g/d
Fibre‡	≥ 20 g/d	≥ 20 g/d	≥ 25 g/d
Protein||	∼15 % total energy	∼15 % total energy	∼15 % total energy
Carbohydrates‡	≥ 50 % total energy	≥ 50 % total energy	≥50 % total energy
Foods¶			
Fruit and vegetables (f&v)**	≥ 5 portions/d	≥ 5 portions/d	≥ 5 portions/d
	1 portion f&v = 50 g	1 portion f&v = 65 g	1 portion f&v = 75 g
	1 portion dried fruit = 20 g	1 portion dried fruit = 25 g	1 portion dried fruit = 30 g
	1 portion of beans/ pulses = 55 g††	1 portion of beans/ pulses = 65 g††	1 portion of beans/ pulses = 75 g††
	1 portion of fruit/vegetable juice or smoothie = 100 ml††	1 portion of fruit/vegetable juice or smoothie = 125 ml††	1 portion of fruit/vegetable juice or smoothie = 145 ml††
Fish total (includes ≥ 1 portion/week of oily fish)	≥ 2 portion/week (≥ 190 g/week)	≥ 2 portion/week (≥ 230 g/week)	≥ 2 portion/week (≥ 270 g/week)
Red/processed meat||	≤ 45 g/d	≤ 55 g/d	≤ 65 g/d

f&v, fruit and vegetables.

* Scientific Advisory Committee on Nutrition (SACN) report on saturated fats and health (2019)^(38)^.

† The Committee on Medical Aspects of Nutrition Policy (COMA) dietary reference value report 1991 states recommendation of ≤ 11 % of energy refers to food energy (excluding alcohol)^(2)^.

‡ Scientific Advisory Committee on Nutrition (SACN) recommendations on carbohydrates, including sugars and fibre (2015), using AOAC fibre^(36)^.

§ Scientific Advisory Committee on Nutrition (SACN) Report on Salt and Health (2003)^(37)^.

|| Protein recommendations obtained from UK Health Security Agency (UKHSA) document ‘From Plate to Guide: What, why and how for the Eatwell model’^(2)^.

¶ Age-appropriate portion sizes defined using previously published methods^(40)^.

** Fruit and vegetables portions include fresh, canned or frozen fruit and vegetables (excluding potatoes)(41).

†† These food groups can only count towards 1 of the 5 portions/d of fruit and vegetables^(41)^.

Monitoring population dietary intake trends over time and assessing the proportion of a population who fail to meet dietary targets can help identify important gaps between actual and recommended intakes. This information is essential for developing targeted public health strategies and policy initiatives to improve a nation’s diet. Data from the National Diet and Nutrition Survey (NDNS), which covers representative samples of the UK population, and UK-based cohort studies, show that on average, UK adults consume too much sugar and saturated fat and not enough fruit and vegetables, fibre and fish^(1,4–6)^, amongst other suboptimal dietary habits. Children and adolescents in the UK are even further off dietary targets: NDNS 2016/17–2018/2019 data show that only 12 % of 11–18-year-olds meet the 5-a-day fruit and vegetable recommendation, only 2 % of 4–10-year-olds and 7 % of 11–18-year-olds limit free sugars to no more than 5 % of total energy intake and only 14 % of 4–10-year-olds and 4 % of 11–18-year-olds meet the fibre recommendations^(7)^.

Establishing healthy dietary patterns during childhood is key since this is when dietary habits are established. Dietary habits often track throughout childhood/adolescence, are key predictors of diet quality later in life^(8–10)^, and affect current and future health status^(11,12)^. There is also clear evidence that diet quality follows a socio-economic gradient in industrialised countries^(13–15)^. For example, children from a lower socio-economic position (SEP) generally consume much fewer fruits and vegetables and more non-core energy-dense nutrient-poor foods^(14)^. These disparities in dietary quality contribute towards the widening health inequalities between socio-economic groups in industrialised countries^(16)^. Understanding how alignment to dietary guidelines in UK children and adolescence differs by factors such as age, sex, SEP and ethnicity could be useful for tailoring public health initiatives.

Dietary quality indices that reflect overall alignment to the package of recommendations covered within dietary guidelines and are useful tools to explore socio-demographic disparities in meeting dietary guidelines. They are also valuable for studying diet quality–disease relationships. However, country-specific dietary guideline indices have mainly been developed for adult populations^(4,17–21)^, with fewer indices designed specifically for use in children^(15,22–26)^. Child-applicable dietary guideline indices need to incorporate age- and sex-specific dietary recommendations from their country. In the UK, a nutrient profile score^(18)^ and an EWG adherence index^(6)^ have been used to assess overall alignment to UK EWG dietary guidelines in adults. To our knowledge, no dietary guideline index based on the UK’s core EWG recommendations has been adapted specifically for use in school-aged children in the UK.

Therefore, this study aimed to (i) create a Children’s Eatwell Guide (C-EWG) score representing overall alignment to UK dietary guidelines for use in school-aged children, by adapting an EWG score previously developed for adults^(6)^, (ii) calculate the proportion of 7–13-year-old children adhering to key UK dietary guidelines using data from the Avon Longitudinal Study of Parents and Children (ALSPAC) and (iii) assess the association between socio-demographic characteristics and the C-EWG score in this cohort.

## Methods

### Study design and sample

This study is based on the index children of ALSPAC, an ongoing multi-generational birth cohort study designed to research how environmental and genetic factors affect people’s health and development across the life course^(27)^. The full methodological details of ALSPAC have been published previously^(28–30)^ and are also available on the ALSPAC website (www.alspac.bris.ac.uk). The initial cohort included 14 541 pregnant women recruited from the South-West of England with expected delivery dates between 1991 and 1992. Of these initial pregnancies, 13 988 children were alive at 1 year. A subsequent recruitment phase^(30)^ in 1999 (child mean age: 7·5 years) resulted in a final sample of 14 673 eligible children. Data were collected on the parents and their children at recruitment and during periodic follow-ups through questionnaires and clinical visits, as well as through medical records. Study data are collected and managed using The Research Electronic Data Capture (REDCap) electronic data capture tools hosted at the University of Bristol^(31)^. The details of all available study data can be found in a fully searchable data dictionary and variable search tool (http://www.bristol.ac.UK/alspac/researchers/our-data/).

### Data collection

Study participant data were collected primarily through self-completed questionnaires, hospital and medical record and periodic face-to-face clinical assessment visits, described in detail previously^(29)^. Maternal pre-pregnancy anthropometric data were collected by self-completed postal questionnaires during pregnancy^(27)^. Maternal educational attainment was recorded as the highest completed out of Certificate of Secondary Education, vocational training, O-level/General Certificate of Secondary Education (qualifications obtained at 16 years of age), A-levels (qualification obtained at 18 years), University degree or higher. Social class was derived using the 1991 Office of Population Censuses and Surveys occupation-based classification, based on the parent’s current or last job at 32 weeks of gestation. This resulted in standardised UK social class classifications: classes I to V (highest–lowest)^(32)^.

### Dietary assessment

Dietary data were collected using 3-d diet diaries, recording all food and drink consumed over two weekdays and one weekend day^(33)^. These were completed prior to research clinic visits when the children were 7, 10 and 13 years of age. The caregiver completed the diaries when the child was 7 years old, while they were completed by the children with assistance from an adult when the children were 10 and 13 years. Portion sizes of foods and drinks consumed were estimated using standard household measures (bowls, cups, teaspoons, dessert spoons, packet size, etc.) and by recording details of volumes of cups/mugs/flasks usually used. A full description of the food and drink consumed was documented with a separate section for description of leftovers. During the clinic visits, the diet diaries were checked by a nutritionist for completeness, discrepancies and clarification of portion sizes. The completed diaries were coded and linked to food composition tables using DIDO (Diet In Data Out). Nutrient intakes were calculated using McCance and Widdowson’s British food composition data^(34)^. Dietary data that were recorded as a mixed dish (i.e. lasagne) within the dietary database was disaggregated into single ingredients if it contained any of the C-EWG foods being analysed. Validity of dietary reporting was calculated using an individualised method based on the ratio of energy intake to estimated energy requirement and its 95 % CI^(35)^.

### UK dietary recommendations

The dietary intakes of the children at 7, 10 and 13 years were compared with current UK dietary recommendations as outlined in the UK Health Security Agency 2016 Report^(3)^ and the nutrient-specific reports that this is based on^(2,36–39)^. Eleven foods and nutrients were assessed: total fat, saturated fat, free sugars, fibre, salt, protein, carbohydrates, fruit and vegetables, non-oily fish, oily fish and red/processed meat. The UK recommended intakes or constraints for each of these foods and nutrients and for the corresponding ages of the participants (7, 10 and 13 years) are detailed in Table 1.

Recommendations for fruit and vegetables, fish and red/processed meat are all specified in terms of number of portions, with portion sizes defined in grams for adults^(2)^. We adjusted the respective portion sizes to child-appropriate portion sizes for the 7-, 10- and 13-year-old children, based on a previously published method^(40)^, described in detail in supplementary materials. Fruit and vegetable recommendations are to eat ≥ 5 portions/d, in accordance with UK 5-a-day guidelines^(2,41)^. Each portion of fresh, canned or frozen fruit and vegetables (excluding potatoes) counts towards the 5-a-day^(41)^. Also included are individual portions of dried fruit. Certain items count only once within a day: these include one portion of fruit juice, vegetable juice or smoothie and one portion of baked beans or legumes^(41)^. The age-specific portion sizes calculated for these subgroups within the 5-a-day are specified within Table 1. Current recommendations for fish intake are to consume at least two portions a week, including at least one portion of oily fish^(42)^. An adult portion of fish is defined as 140 g^(2)^, which was adjusted to child-appropriate portions (Table 1) as described in the Supplementary Methods section. It is recommended that red and processed meat intake are limited to ≤ 70 g/d for adults^(2)^, which was similarly adjusted to child-appropriate portions (Table 1).

### Summary Children’s Eatwell Guide score

The C-EWG score is the sum of points assigned according to adherence to guidelines for total fat, saturated fat, free sugars, fibre, salt, fruit and vegetables, non-oily fish, oily fish and red/processed meat (it does not include carbohydrates or protein), in line with previous methods^(6)^. Since fish guidelines can be broken down into recommendations for consumption of oily fish and non-oily fish, these are included separately within the C-EWG score^(6)^. Each participant’s intake of the nine foods and nutrients was dichotomised into adhering to (1 point) or not adhering to (0 points) the dietary guidelines at each age. The foods and nutrients were examined separately and as a summary C-EWG score, representing how many of nine recommendations were met by each participant. Each food and nutrient contributed an equal weight within the score. The C-EWG scores ranged from 0 to 9 (none to all recommendations met).

### Sensitivity analyses

For total fat, saturated fat and free sugars dietary guidelines specify intakes as a percentage of total energy^(36,38)^ as analysed above and also as age- and sex-specific recommendations in grams per day^(3)^. Therefore, in sensitivity analysis, we assessed adherence to guidelines for these three nutrients using g/d as well^(3)^. Intake of free sugars was also assessed using the previous recommendation of ≤ 10 % of total energy set by the Food Standards Agency before the new reduced free sugar guidelines were introduced in 2014–2015^(36)^. This is relevant because these higher sugar limits would have been in place when the dietary assessments at 7, 10 and 13 years were carried out.

### Statistical analysis

Complete dietary data were available for 7262 children at 7 years, 7449 children at 10 years and 6094 children at 13 years. However, the proportion of children classified as dietary under-reporters increased substantially from 7 years (12 %) to 13 years (62 %) (online Supplementary material Fig. 1). Therefore, to obtain more accurate intake estimates, the main analysis was restricted to plausible dietary reporters. In additional analyses presented in Supplementary material, adherence to each of food and nutrient recommendation in the main analysis of valid dietary reporters was compared with adherence in the full cohort with dietary data (under-reporters, over-reporters and plausible reporters). In addition, the baseline characteristics of the eligible cohort with incomplete or implausible dietary data were compared with the final sample of plausible dietary reporters. For all participants classified as plausible dietary reporters, their dietary intake at each age was described with means (sd). Data on intake of food groups were mainly non-parametric, so medians (interquartile range) were also calculated. The percentage of children meeting different numbers of C-EWG recommendations was calculated at each age using the C-EWG score. The average percentage deviance from each EWG recommendation at each age was calculated using the mean intake for each food and nutrient. The association between socio-demographic characteristics (sex, ethnic background, maternal highest educational attainment, family social class and age of mother at delivery) and child and maternal anthropometric characteristics (BMI of child at age of dietary data collection and pre-pregnancy BMI of mother) was analysed according to the C-EGW score (continuous variable) at 7, 10 and 13 years using multivariable linear regression models, mutually adjusted for all covariates. These variables were selected a priori, based on previous literature on socio-demographic predictors of dietary quality in children^(14,16)^. All statistical analyses were performed using Stata version 15.1 (Stata Corporation).

## Results

**Fig. 1.**
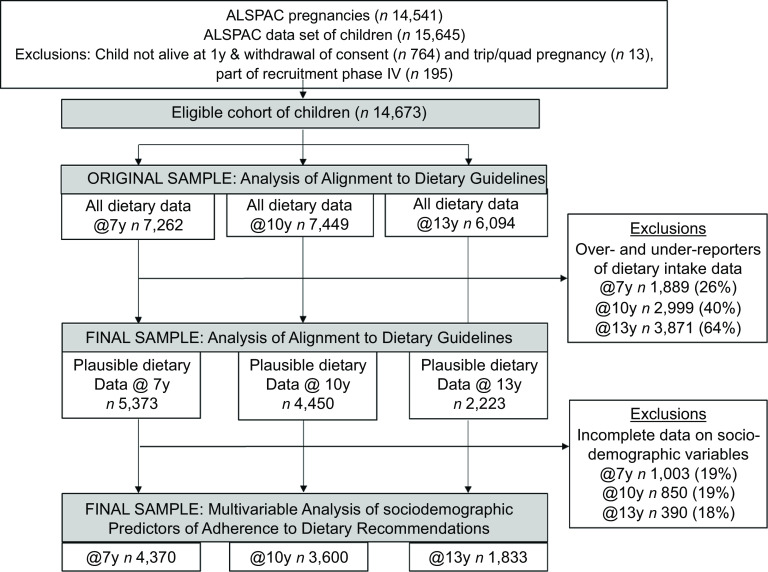
Study flow diagram of participants from the Avon Longitudinal Study of Parents and Children (ALSPAC). The present study uses data from participants with complete plausible dietary data at 7, 10 and 13 years and complete data on socio-demographic characteristics.

Of the baseline cohort of 14 673 eligible participants, complete and valid dietary data were available for 5373 children at 7 years (mean age: 7·5 years (sd = 0·3)), 4450 children at 10 years (mean age: 10·6 years (sd = 0·2)) and 2223 children at 13 years (mean age: 13·8 years (sd = 0·2)) (Fig. 1). Compared with the initial eligible sample, the final samples included in our analyses were more likely to have a higher family social class, a mother with a higher educational attainment and a lower BMI and the child was more likely to have a lower BMI at the time of dietary data collection (online Supplementary Tables 1a-b).

### Adherence to dietary recommendations – nutrients

Table 2 details the dietary intakes of the seven nutrients, for the whole cohort and stratified by the children’s alignment to dietary recommendations. Sixty percent of the children consumed over the recommended limit for total fat intake at 7 years, 63·7 % at 10 years and 60·5 % at 13 years. A larger proportion of the cohort consumed over the recommended upper limit for percentage of energy from saturated fat: 90·3 % of 7-year-olds, 87·9 % of 10-year-olds and 81·3 % of 13-year-olds. For free sugars, most of the cohort failed to meet the recommendation of free sugar forming ≤ 5 % of total energy: approximately 99 % at all three ages. Accordingly, the overall mean percentage of energy from free sugars was approximately three times above the recommendation. The proportion of children consuming over the recommended limit of salt was 71·4 % at 7 years, 89·0 % at 10 years and 81·5 % at 13 years. A large proportion of the children failed to consume the recommended daily amount of dietary fibre: 92·3 % at 7 years, 81·5 % at 10 years and 83·3 % at 13 years. The proportion of the cohort not adhering to the guidance for protein was 84·1 % at 7 years, 81·8 % at 10 years and 83·3 % at 13 years. In contrast, recommendations for carbohydrate intake were better adhered to with only 22·7 %, 27·1 % and 31·4 % not meeting the guidance at 7, 10 and 13 years, respectively.

**Table 2.** Intake of key nutrients within the Eatwell Guide by children at 7, 10 and 13 years of age from the Avon Longitudinal Study of Parents and Children (ALSPAC), according to adherence to the UK dietary guidelines

		ALSPAC children at 7 years (*n* 5373)			ALSPAC children at 10 years (*n* 4450)			ALSPAC children at 13 years (*n* 2223)		
UK dietary recommendations for key nutrients		%	Mean	sd	%	Mean	sd	%	Mean	sd
Total fat, % total energy (≤ 35 % total energy)*	All	100·0	36·1	4·4	100·0	36·6	4·7	100·0	36·3	5·3
	Not Adherent	60·4	39·0	2·9	63·7	39·4	3·1	60·5	39·7	3·5
	Adherent	39·6	31·8	2·6	36·3	31·7	2·8	39·5	31·2	3·1
Saturated fat, % food energy (≤ 11 % food energy)*,†	All	100·0	14·5	2·8	100·0	14·2	2·9	100·0	13·9	3·3
	Not Adherent	90·3	15·0	2·5	87·9	14·8	2·5	81·3	14·9	2·6
	Adherent	9·7	9·8	1·0	12·2	9·8	1·1	18·7	9·5	1·4
Free sugars, % total energy	All	100·0	17·2	5·1	100·0	17·8	5·4	100·0	17·5	6·1
(≤ 5 % total energy)‡	Not Adherent	99·9	17·2	5·0	99·6	17·8	3·6	99·2	17·6	5·9
	Adherent	0·1	4·1	0·8	0·4	3·6	1·0	0·8	3·4	1·2
(≤ 10 % total energy)§	Not Adherent	94·2	17·8	4·7	93·8	18·4	4·9	91·2	18·5	5·4
	Adherent	5·8	8·4	1·4	6·2	8·1	1·7	8·8	7·7	1·9
Salt, g/d (≤ 5 g/d at 7 and 10 years, ≤ 6 g/d at 13 years)||	All	100·0	5·8	1·3	100·0	6·9	1·7	100·0	8·0	2·2
	Not Adherent	71·4	6·4	1·0	89·0	7·2	1·5	81·5	8·6	1·9
	Adherent	28·6	4·4	0·5	11·0	4·5	0·5	18·5	5·3	0·7
Fibre, g/d (≥ 20 g/d at 7 years and 10 years ≥ 25 g/d at 13 years)‡,¶	All	100·0	13·9	4·1	100·0	16·1	4·7	100·0	19·4	6·4
	Not Adherent	92·3	13·1	3·2	81·5	14·5	3·1	83·3	17·3	4·0
	Adherent	7·7	22·8	2·9	18·5	23·5	3·3	16·7	30·2	4·8
Protein, % total energy, (≥ 15 % total energy)**	All	100·0	13·0	2·1	100·0	13·1	2·3	100·0	13·5	2·5
	Not Adherent	84·1	12·4	1·5	81·8	12·3	1·6	75·3	12·4	1·7
	Adherent	15·9	16·5	1·4	18·2	16·6	1·6	24·7	16·7	1·5
Carbohydrate, % total energy (≥ 50 % total energy)‡	All	100·0	53·6	4·9	100·0	53·1	5·1	100·0	52·9	5·8
	Not Adherent	22·7	47·2	2·6	27·1	46·8	2·7	31·4	46·4	3·3
	Adherent	77·3	55·5	3·7	72·9	55·4	3·7	68·6	55·8	4·1

* Scientific Advisory Committee on Nutrition (SACN) report on saturated fats and health (2019)^(38)^.

† The Committee on Medical Aspects of Nutrition Policy (COMA) Dietary reference value report 1991 states recommendation of ≤ 11 % of energy refers to food energy (excluding alcohol)(3,39).

‡ SACN recommendations on carbohydrates, including sugars and fibre (2015)^(36)^.

§ Sugar recommended intake at ≤ 10 % of total energy according to Food Standards Agency guidelines before 2013^(36)^.

|| Scientific Advisory Committee on Nutrition (SACN) Report on Salt and Health (2003)^(37)^. Dietary data on Na intake were converted to salt intake by multiplying by 2·54.

¶ The non-starch polysaccharide (NSP) fibre intakes recorded in the ALSPAC data set were converted into AOAC fibre equivalent values using the standard conversion factor of 1·33.

** Protein recommendations obtained from UKHSA document ‘The Eatwell Guide’^(2)^.

### Adherence to dietary recommendations – foods

**Fig. 2.**
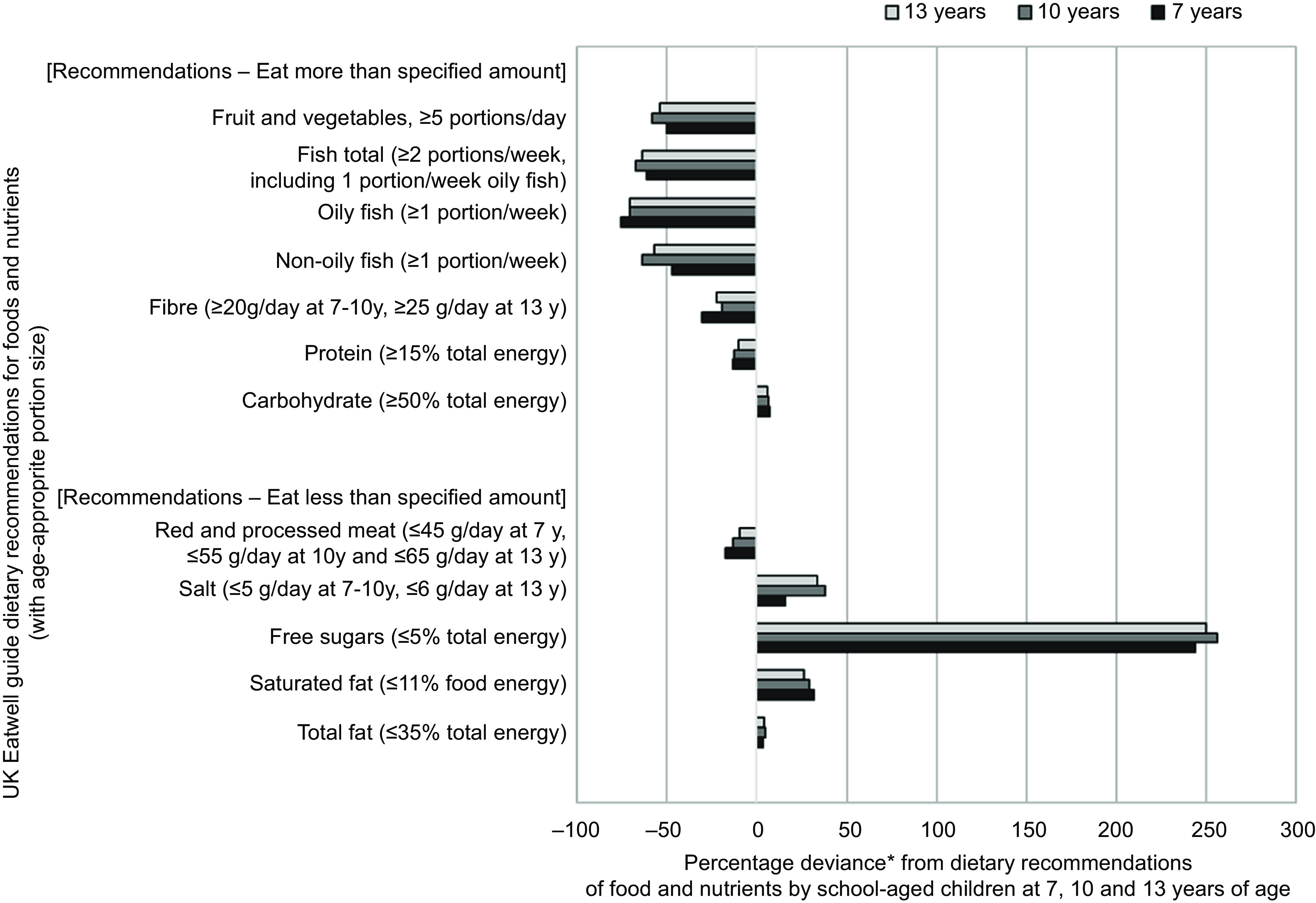
Percentage deviance from UK dietary recommendations by Avon Longitudinal Study of Parents and Children (ALSPAC) children at 7 years (*n* 5373), 10 years (*n* 4450) and 13 years (*n* 2223) of age. Footnotes: *Percentage deviance calculation: ((mean intake - recommended minimum intake or limit)/recommended minimum intake or limit) × 100. 0 % reflects no deviance: mean intake is on the cut-off defined by recommendations

Table 3 details the dietary intakes for fish, fruit and vegetables and red and processed meat, for the whole cohort and stratified by participants’ adherence. At all three ages, approximately a third of children consumed more than the recommended amount of red and processed meat. Most of children failed to meet the recommendations for total fish (non-oily and oily fish): 94·1 % at 7 years, 93·5 % at 10 years and 94·0 % at 13 years. At all ages, a greater proportion of children met recommendations for non-oily fish compared with oily fish: 21·6 % for non-oily fish *v*. 9·5 % for oily fish at 7 years. For the 5-a-day fruit and vegetable recommendation, 84·8 % of 7-years-olds, 91·8 % of 10-year-olds and 88·3 % of 13-year-olds failed to meet these guidelines. The mean number of portions per day was 2·5 at 7 years, 2·1 at 10 and 2·0 at 13 years. The greatest percentage deviance from EWG food and nutrient recommendations was for sugar intake, followed by fish and then fruit and vegetable intake (Fig. 2).

**Table 3.** Intake of key foods within the Eatwell Guide by children at 7, 10 and 13 years of age from the Avon Longitudinal Study of Parents and Children (ALSPAC), according to adherence to the UK dietary guidelines

UK Eatwell Guide Key Food Groups*	Adherence to guidelines	ALSPAC children at 7 years (*n* 5373)					ALSPAC children at 10 years (*n* 4450)					ALSPAC children at 13 years (*n* 2223)				
		*%*	Mean	sd	Median	IQR	*%*	Mean	sd	Median	IQR	*%*	Mean	sd	Median	IQR
Red and processed meat, g/d	All	100·0	37·1	29·0	32·3	15·6–52·7	100·0	47·6	37·5	42·0	20·0–67·2	100·0	58·8	50·5	50·0	20·9–83·9
	Not Adherent	32·7	70·2	23·3	63·8	53·1–80·7	36·2	86·9	30·6	77·9	65·0–98·5	38·4	108·6	43·8	95·6	77·6–125·0
	Adherent	67·3	21·1	14·0	21·9	10·0–32·8	63·8	25·3	17·4	25·7	10·4–40·2	61·6	27·7	20·9	27·2	7·3–45·5
Fish total, g/week† (≥ 2 portions/week, incl. 1 portion/week oily fish)	All	100·0	73·4	125·6	0·0	0·0–119·4	100·0	74·9	147·3	0·0	0·0–116·7	100·0	97·5	207·0	0·0	0·0–130·0
	Not Adherent	94·1	56·0	99·6	0·0	0·0–198·9	93·5	51·8	108·3	0·0	0·0–81·7	94·0	70·3	156·7	0·0	0·0–105·0
	Adherent	5·9	353·4	163·7	315·0	243·0–408·3	6·5	405·9	218·6	336·7	268·3–450·9	6·0	524·5	370·3	420·0	338·3–571·6
Non-oily fish, g/week† (≥ 1 portions/week)	All	100·0	50·0	100·3	0·0	0·0–79·7	100·0	41·4	106·4	0·0	0·0–0·0	100·0	58·1	151·8	0·0	0·0–0·0
	Not Adherent	78·4	10·1	25·1	0·0	0·0–0·0	84·3	6·4	22·1	0·0	0·0–0·0	84·0	7·6	27·2	0·0	0·0–0·0
	Adherent	21·6	194·9	132·3	154·0	119·6–218·4	15·7	228·3	166·7	172·1	121·3–239·1	16·0	323·8	237·7	227·5	172·3–389·7
Oily fish, g/week† (≥ 1 portions/week)	All	100·0	23·5	76·0	0·0	0·0–0·0	100·0	33·6	100·4	0·0	0·0–0·0	100·0	39·4	143·3	0·0	0·0–0·0
	Not Adherent	90·5	3·2	15·6	0·0	0·0–0·0	89·0	5·3	20·9	0·0	0·0–0·0	89·8	5·3	22·7	0·0	0·0–0·0
	Adherent	9·5	216·4	131·8	175·0	116·7–261·3	11·0	262·6	171·0	231·0	157·7–312·7	10·2	340·2	311·8	245·0	198·3–373·3
Fruit and vegetables, g/d‡ (≥ 5 portions/d)	All	100·0	141·3	95·1	126·0	70·8–194·3	100·0	150·1	104·3	134·4	76·3–205·7	100·0	181·4	130·8	158·3	89·0–246·3
	Not Adherent	84·8	112·1	63·5	109·6	62·7–158·5	91·8	129·9	78·3	125·0	70·3–186·3	88·3	147·4	90·0	144·3	80·0–210·0
	Adherent	15·2	304·1	76·2	286·8	253·7–340·3	8·3	374·5	94·0	351·2	308·7–420·7	11·7	437·1	103·2	421·2	357·7–496·7
All, portions/d	All	100·0	2·5	2·0	2·0	1·0–4·0	100·0	2·1	1·7	2·0	1·0–3·0	100·0	2·3	1·8	2·0	1·0–3·0
Fruit, g/d	All	100·0	79·3	75·4	66·7	20·0–117·3	100·0	71·1	76·3	53·2	0·0–103·3	100·0	86·4	98·0	66·7	0·0–133·3
Vegetables, g/d	All	100·0	52·3	42·3	44·3	21·0–74·0	100·0	66·2	56·3	57·0	23·0–96·7	100·0	82·3	70·0	72·2	29·3–119·7
Pulses, g/d	All	100·0	8·7	14·8	0·0	0·0–12·8	100·0	11·5	21·7	0·0	0·0–17·5	100·0	10·9	23·6	0·0	0·0–13·3
Dried fruit, g/d	All	100·0	0·9	4·0	0·0	0·0–0·0	100·0	1·2	5·3	0·0	0·0–0·0	100·0	1·7	6·6	0·0	0·0–0·0
Fruit/veg juice/smoothie, ml/d	All	100·0	82·8	125·1	17·5	0·0–131·6	100·0	119·8	151·7	62·7	0·0–188·0	100·0	178·6	212·4	125·3	0·0–282·0

* Age appropriate portion sizes defined using previously published methods^(40)^.

† Fish recommendations according to Scientific Advisory Committee on Nutrition (SACN) report on fish consumption.

‡ Fruit and vegetables portions include fresh, canned or frozen fruit and vegetables (excluding potatoes) and dried fruit, such as currants, dates, sultanas and figs^(41)^. A portion of fruit/vegetable juice or smoothie and one portion of baked beans and legumes count only once^(41)^. Fruit and vegetables g/d for all, not adherent and adherent do not include fruit/vegetable juice or smoothies.

**Fig. 3.**
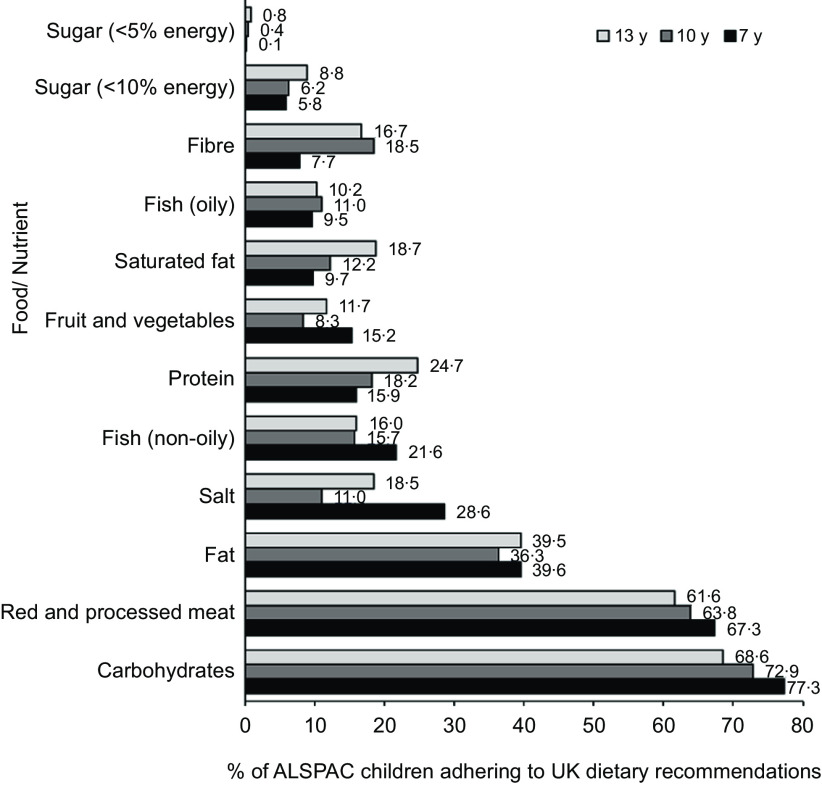
Percentage of Avon Longitudinal Study of Parents and Children (ALSPAC) participants adhering to UK dietary recommendations at 7 years (*n* 5373), 10 years (*n* 4450) and 13 years (*n* 2223).

Figure 3 illustrates the percentage of the ALSPAC children meeting the UK dietary recommendations for the different C-EWG foods and nutrients at 7, 10 and 13 years. Although the proportion of children meeting each guideline changed from 7 to 13 years (increasing or decreasing depending on the food/nutrient), in general a similar overall pattern was observed at 10 and 13 years.

### Children’s Eatwell Guide score dietary recommendations

The mean C-EWG score at 7 years was 2·0 (sd 1·3), at 10 years it was 1·8 (sd 1·3) and at 13 years it was 1·9 (sd 1·4), ranging from 0–7 at all ages (maximum possible score of 9). The C-EWG score showed that 12·1 % of the participants at 7 years did not meet any of the nine UK dietary recommendations included in the score, while approximately a quarter (26·9 %) met one recommendation, 28·2 % met two recommendations and a third (32·7 %) met three or more recommendations (Table 4). Only a small proportion (12·7 %) of children at 7 years met four or more recommendations; 8·6 % met 4 recommendations, 3·3 % met 5 recommendations, less than 1 % met 6 or 7 recommendations and none of the children met all 9 recommendations (data not tabulated). In general, a similar pattern was seen at 10 and 13 years, with the majority of children at each age (∼70 %) meeting fewer than three of the nine C-EWG recommendations analysed.

**Table 4.** Percentage of individual recommendations met by children at 7, 10 and 13 years of age, according to number of recommendations met in the Children’s Eatwell Guide (C-EWG) score

Percentage of participants meeting recommendations for foods and nutrients	Children’s Eatwell Guide (C-EWG) score: number of recommendations met			
	Zero (%)	One (%)	Two (%)	≥ Three (%)
At 7 years, all (*n* 5373)	12·1	26·9	28·2	32·7
Total fat	0·0	15·9	39·0	74·3
Saturated fat	0·0	0·8	3·4	26·2
Free sugars	0·0	0·1	0·1	0·3
Salt	0·0	10·2	21·3	51·3
Fibre	0·0	1·5	4·2	18·7
Red and processed meat	0·0	53·7	81·3	91·4
Oily fish	0·0	3·0	8·4	19·3
Non-oily fish	0·0	9·3	21·2	40·0
Fruit and vegetables	0·0	5·5	10·2	33·0
				
At 10 years, all (*n* 4450)	15·4	30·9	28·0	25·7
Total fat	0·0	14·4	45·3	74·6
Saturated fat	0·0	1·3	7·3	38·0
Free sugars	0·0	0·2	0·6	0·7
Salt	0·0	2·4	14·4	24·2
Fibre	0·0	7·3	18·2	43·3
Red and processed meat	0·0	61·5	78·0	89·4
Oily fish	0·0	4·4	12·4	24·1
Non-oily fish	0·0	7·0	17·7	33·6
Fruit and vegetables	0·0	1·7	6·2	23·4
				
At 13 years, all (*n* 2223)	14·9	27·5	26·0	31·6
Total fat	0·0	16·5	39·1	78·5
Saturated fat	0·0	1·0	12·3	48·1
Free sugars	0·0	0·7	1·7	0·6
Salt	0·0	5·9	23·0	34·4
Fibre	0·0	6·6	13·8	35·7
Red and processed meat	0·0	54·3	73·7	86·9
Oily fish	0·0	3·3	12·6	18·9
Non-oily fish	0·0	9·0	15·2	30·2
Fruit and vegetables	0·0	2·8	8·5	27·7

### Children’s Eatwell Guide score and socio-demographic factors

The association between the C-EWG score at each age and the mutually adjusted children’s socio-demographic characteristics is shown in Table 5. At 13 years, being female was weakly associated with a higher C-EWG score compared with being male, but there was no evidence of sex differences at the other ages. Being from other ethnic groups compared with white ethnic group was associated with having a higher C-EWG score at 7 and 10 years old but not at 13 years. Lower maternal education and lower household social class were associated with lower C-EWG scores at all ages. Maternal education was one of the strongest factors associated with lower C-EWG scores at 10 years (*ß* − 0·39 (95 % −0·51, −0·27) for low *v*. high educational attainment). The child’s BMI at the time of dietary data collection was associated with a lower C-EWG score at 7 years but not at other ages. Likewise, maternal overweight/obesity was associated with the child having a lower C-EWG score at 7 and 10 years. A younger maternal age at delivery was only associated with a lower EWG score at 7 years.

**Table 5.** Association between socio-demographic characteristics and Children’s Eatwell Guide (C-EWG) score in children at 7, 10 and 13 years of age

Socio-demographic characteristics of the ALSPAC children	C-EWG Score at 7 years (*n* 4370)				C-EWG Score at 10 years (*n* 3600)					C-EWG Score at 13 years (*n* 1833)		
	Total *n*	*ß* *	95 % CI	*P*-value	Total *n*	*ß* *	95 % CI	*P*-value	Total *n*	*ß* *	95 % CI	*P*-value
Sex												
Male	2221	Reference			1779	Reference			885	Reference		
Female	2149	0·05	–0·03,0·13	0·192	1821	–0·04	–0·13,0·05	0·368	948	0·16	0·00,0·31	0·044
Child ethnic background												
White	4234	Reference			3489	Reference			1772	Reference		
Other than white	136	0·34	0·12,0·56	0·002	111	0·41	0·18,0·65	0·001	61	–0·18	–0·53,0·17	0·312
Child BMI z-score category												
Normal weight	3838	Reference			3226	Reference			1684	Reference		
Overweight and obese	532	–0·15	–0·28,–0·03	0·013	374	0·06	–0·09,0·20	0·425	149	–0·10	–0·34,0·15	0·438
Energy intake (kJ/d)												
Per 1000 kJ increment	4370	–0·19	–0·23,–0·15	< 0·001	3600	–0·08	–0·12,–0·04	< 0·001	1833	–0·06	–0·11,–0·01	0·011
Maternal highest education attainment												
A level and degree	1978	Reference			1651	Reference			868	Reference		
Vocational or O level	1575	–0·11	–0·21,–0·02	0·017	1299	–0·22	–0·32,–0·12	< 0·001	650	–0·25	–0·40,–0·09	0·002
Secondary education (CSE)	817	–0·23	–0·34,–0·11	< 0·001	650	–0·39	–0·51,–0·27	< 0·001	315	–0·28	–0·47,–0·08	0·005
Highest household social class												
I and II	1334	Reference			1086	Reference			574	Reference		
III non-manual and manual	2333	–0·22	–0·32,–0·13	< 0·001	1937	–0·14	–0·25,–0·04	0·006	981	–0·20	–0·36,–0·05	0·012
IV and V	703	–0·24	–0·37,–0·12	< 0·001	577	–0·10	–0·23,0·04	0·178	278	–0·37	–0·59,–0·16	0·001
Maternal BMI category												
< 25 kg/m^2^	3551	Reference			3007	Reference			1551	Reference		
≥ 25 kg/m^2^	819	–0·12	–0·22,–0·02	0·016	593	–0·16	–0·27,–0·04	0·006	282	–0·09	–0·27,0·08	0·301
Maternal age at delivery, years												
≥ 30	2060	Reference			1669	Reference			857	Reference		
16–29	2310	–0·14	–0·22,–0·06	< 0·001	1931	–0·03	–0·12,0·05	0·435	976	–0·05	–0·18,0·08	0·469

ALSPAC, Avon Longitudinal Study of Parents and Children; CSE, Certificate of Secondary Education.

* Multivariable regression models, mutually adjusted for all covariates listed in the table at each age.

### Sensitivity analyses

A greater proportion of children were classified as meeting the dietary guidelines for fat and saturated fat at 7 years when using the recommendations specifying sex- and age-specific g/d compared with percentage of dietary energy, while at 13 years there were minimal differences (online Supplementary Table 2). There were only small differences in classification of children meeting and not meeting guidelines between the two definitions for free sugars.

Supplementary Fig. 2 illustrates the percentage of children meeting each food and nutrient dietary recommendation for the whole cohort compared with the plausible dietary reporters only which are presented in the main analysis. At 10 and 13 years, the proportion of children meeting dietary recommendations for total fat, saturated fat, salt, free sugars, protein and red and processed meat was lower in the plausible diet reporting group. These differences were generally more pronounced at 13 years.

## Discussion

This study created a C-EWG score to represent overall alignment to UK dietary guidelines in children using age-appropriate portion sizes and to provide a relevant assessment of dietary quality. The findings indicate that the dietary intakes of school-aged children in this population-based UK birth cohort were mostly suboptimal compared with national dietary recommendations. Overall, their diets contained insufficient fruit and vegetables, fibre, fish and excessive amounts of salt, total and saturated fat and sugar, relative to recommendations. Approximately 90 % of the children did not meet fish or fibre guidelines, 99 % of the children were over the recommended sugar limit and 85–90 % did not meet the 5-a-day fruit and vegetable recommendation. However, over half the cohort met the recommendations for carbohydrate intake and limiting red/processed meat. Of particular concern is that 12–15 % of the 7–13-year-old children did not meet any of the nine C-EWG score recommendations and approximately a third met only one recommendation. This implies that for the majority of children in this cohort their dietary habits did not comply with key government dietary recommendations, which is not unexpected given the prevalence of dietary-related chronic diseases in the UK. Meeting fewer UK dietary recommendations during childhood (lower C-EWG scores) was associated with a lower social class, lower maternal educational attainment and maternal characteristics such as being overweight/obese and a younger age in pregnancy.

The overall poor alignment to UK dietary guidelines by the children in this study aligns with previous studies reporting large discrepancies between dietary habits and national dietary guidelines in similarly aged children from Australia^(15,43)^, the USA^(44)^, the Netherlands^(24)^ and Malaysia^(45)^. An Australian study of a nationally representative sample of 789 4–8-year-olds found that 95 % did not meet vegetable recommendations, 81 % were above the saturated fat recommended limits and 72 % were above the sodium recommended limits^(43)^. A nationally representative dietary survey of 3688 9–13-year-olds in the USA also found that most children did not meet government recommendations for the nutrient-rich food groups (except total grains and meat and beans) and exceeded energy intakes from solid fats and added sugar^(44)^. A population-based cohort study of 4733 8-year-old children in the Netherlands constructed a diet quality score based on Dutch dietary guidelines^(24)^ and also reported that the proportion of children failing to meet guidelines was high for many key foods and nutrients: 84 % for vegetables, 70 % for fruit, 64 % for fish, 97 % for fats and 98 % for sugar-containing beverages.

To our knowledge, no prior research has assessed overall adherence to EWG dietary recommendations in UK school children, although suboptimal intakes of individual foods and nutrients have been reported^(46–48)^. Adherence to overall dietary recommendations has been studied in adults living in the UK^(4–6)^. The study which the C-EWG score was adapted from reported that approximately 10 % of their nationally representative NDNS adults met only 0–1 of the nine recommendations, while 60 % adhered to at least three recommendations^(6)^. These findings together with population-based cohort studies indicate that a large proportion of adults in the UK fail to meet dietary guidelines for fibre, saturated fat, sugar, fish and fruit and vegetables, in line with our study of UK school children^(5,6)^. This is not surprising since poor dietary habits that are established during childhood/adolescence often become embedded and then track into adulthood^(8,10)^.

Our research, along with the findings from other studies in industrialised countries, highlights the overabundance of saturated fat, sugar and salt in most UK children’s diets. This is likely to be the result of the targeted marketing, availability, affordability and palatability of processed cereal-based baked products, salty snack foods, confectionary and sugar-sweetened beverages^(35)^. Meeting UK population targets for free sugars is particularly challenging, and in the 2016/2017–2018/2019 NDNS survey data only 2 % of 4–10-year-olds consumed ≤ 5 % of energy from free sugars, similar to the very low percentages observed in the 7–13-year-olds in our study (0·4–0·8 %). However, the UK soft drinks industry levy, announced in 2016 and implemented in 2018, may have had some positive impact on sugar consumption: 1 year after its implementation there was a reported decrease of around 30 g of purchased sugar in soft drinks per household per week^(49)^.

Approximately two-thirds of the children in the cohort exceeded the recommended upper limit of salt intake, which is in line with findings from a cross-sectional study of children in London from 2007 to 2010^(47)^. In their study, they reported key food sources of salt were processed foods, above all cereal-based products and meat products. A national salt reduction programme was implemented in 2003/2004 by the UK Food Standards Agency along with Consensus Action on Salt & Health, and NDNS data show there has been a reduction in salt intake from foods from 2008/2009–2016/2017 in all age groups, with –1·25 g/d of salt for children^(50)^.

Our findings indicate that fish consumption was well below the guidelines in all age groups, with only 6 % of the 7–13-year-olds eating at least 2 portions of fish a week (at least one being oily fish). In addition, oily fish consumption was approximately half of that of non-oily fish. NDNS 2014/2015–2015/2016 data on mean intakes of total fish (12 g/d) and oily fish (2 g/d) in 4–10-year-olds are similar to the range of intakes in the 7–13-year-olds in our study^(51)^. Fish, especially oily fish, are a rich source of *n*-3 long-chain polyunsaturated fats, which are important for optimal neurocognitive development early in life. Therefore, the large gap between actual and recommended intakes for fish is of particular public health concern.

Approximately 90 % of the 7–13-year-olds in our study did not meet the 5-a-day recommendation for fruits and vegetables, averaging 2·1 to 2·5 portions/d instead. NDNS 2014–2016 reports on 11–18-years-olds showed that 92 % failed to meet 5-a-day recommendations, eating an average of 2·7 portions/d^(51)^, which is not dissimilar to our results. The issue of children’s low consumption of fruits and vegetables (particularly vegetables) relative to guidelines is common to many industrialised countries^(24,43,44)^. This is of particular concern because fruit and vegetables are important sources of dietary vitamins, minerals, antioxidants and fibre which are needed for adequate growth and development and to support children’s immune systems to help fight illnesses^(46)^. A diet rich in fruit and vegetables also reduces the risk of developing chronic diseases later in life, such as CVD, diabetes, obesity and some types of cancers^(46,52)^. Therefore, it is especially important to establish sufficient daily consumption of fruit and vegetables from childhood. Schools are useful targets to improve children’s dietary quality: the School Fruit and Vegetable Scheme offered to Reception and Key Stage 1 schoolchildren (∼4–7 years) in the UK is an initiative launched in 2004 to try and increase consumption of fruit and vegetables in children.

Fibre intake in our study was well below dietary guidelines, with only 8 %, 19 % and 17 % of children meeting the recommended intake at 7-, 10- and 13 years. These figures are comparable to those reported by studies in UK adults using dietary data from the UK Biobank^(4)^ (10·9 %) and NDNS ^(5)^ (18 %). The fibre intake of 13·9 g/d (sd 4·1) in 7-year-olds in our study was also similar to the mean intake of 14·3 g/d (sd 4·5) in 4–10-year-old children in the NDNS 2016/2017–2018/2019^(53)^. Trend analyses using NDNS data have shown that fibre intakes within the UK have barely changed over the 11 years since 2008 ^(53,54)^. This is despite numerous public health campaigns and initiatives since 2015 aimed at increasing fibre intake ^(36)^.

In terms of predictors of dietary quality in children, our findings showed that a lower family social class and several maternal characteristics were associated with meeting fewer UK dietary recommendations during childhood, in line with previous literature^(13,15,16)^. Lower maternal education was one of the strongest independent predictors of poor compliance to dietary guidelines, consistent with a population-based cohort of children in the Netherlands^(24)^. A systematic review of socio-economic disparities in diet among adolescents and young adults also concluded that a higher SEP, above all higher educational attainment, was associated with better dietary quality scores, higher intakes of fruits and vegetables and dairy products and lower intakes of energy-dense foods and sugary sweetened beverages^(16)^. A higher level of parental education is also linked to better nutritional knowledge^(13)^. According to a systematic review of SEP and predictors of children’s dietary intake, SEP was strongly related to children’s nutrition knowledge, parental modelling, home food availability and accessibility^(14)^.

Our study found that children with ethnic backgrounds other than white had diets which were more closely aligned to EWG dietary recommendations, similar to findings from a study in the UK Biobank (in women)^(4)^. Likewise, in a cohort in the Netherlands, the children with Moroccan ethnic background had a higher dietary quality compared with those with Dutch ethnicity, which was due to maintaining a more Mediterranean-style diet^(24)^. In *post hoc* analyses, we found that ALSPAC children with other than white ethnicity consumed less red/processed meat and more fish, fruit, vegetables and legumes, which could be due to ethnic-specific retention of traditional diets, irrespective of SEP.

Finally, we observed that maternal overweight/obesity was associated with a lower EWG score in children at 7 and 10 years, suggesting that an unhealthy lifestyle of the mother (poor dietary habits and/or lack of physical activity), may have had a negative influence on children’s dietary quality, which has been shown in previous research^(24)^. Understanding the determinants of poorer dietary quality in UK children can be used to target dietary interventions at high-risk groups.

In terms of the study limitations, the dietary intakes reported may not be generalisable to the UK general population of this age group. Although the ALSPAC children were relatively representative of the population in the study area at birth^(28)^, sample attrition during the 13-year follow-up for this analysis introduced some follow-up bias. In particular, the sub-population of the cohort included in our analysis were more likely to be from a higher SEP. Furthermore, previous research within ALSPAC showed that a posteriori unhealthy dietary patterns correlated with several socio-economic factors^(55)^. Since children from lower SEP and therefore with poorer quality diets were under-represented in our analysis, the proportion of children aligning to dietary recommendations might be even lower in a representative sample of the UK children than in our study. The dietary data analysed are also not contemporary: diet diaries were recorded between 1998–2000 for the 7-year-olds, between 2002–2003 for the 10-year-olds and between 2005–2006 for the 13-year-olds. However, trends in dietary intakes of 4–10-year-olds using NDNS data showed that from 2008/2009 to 20018/2019 there were no significant changes in consumption of total and saturated fat, fruit and vegetables or fibre^(50)^. In contrast, there was a decrease in percentage of energy from free sugars and red and processed meat and salt intake^(50)^. Another consideration is that several EWG recommendations were different or not in place when the dietary data were collected. For instance, the limit for percentage of energy from free sugars was ≤ 10 % at the time of data collection, and then it was decreased to ≤ 5 % in 2015 ^(36)^. Therefore, in sensitivity analyses, alignment to the previous guidelines was also assessed and showed similar results. The 5-a-day fruit and vegetables recommendation was launched in March 2003, so would not have been in place when the dietary data were collected for the 7–10-year-olds.

A strength of this study is that to reduce recall bias we restricted our analysis to plausible dietary reporters, since there was a substantial proportion of under-reporters, particularly at 13 years of age. Sensitivity analyses comparing all participants with dietary data to the final sample with plausible dietary data revealed differential under-reporting for certain foods and nutrients, in particular greater under-reporting of the more socially undesirable foods (foods high in fat, salt and sugar), which is a common feature of dietary reporting bias ^(56)^. Mixed dishes were disaggregated into single ingredients to allow a more precise estimation of dietary intake of EWG foods. The adult portion sizes for the EWG food groups analysed were modified to age-appropriate portion sizes for children using a method which can be adapted for different ages of children in future studies. We were also able to assess compliance to dietary guidelines and predictors of dietary compliance at three separate points throughout childhood.

In conclusion, there were large discrepancies between the dietary intakes of school-aged children in this cohort and UK dietary guidelines, especially in children from lower SEP. In general, the poorest alignment to guidelines was for fibre, saturated fat, sugar, salt and fruit and vegetables. Unhealthy dietary habits can negatively impact growth and development during childhood and are an important risk factor contributing to the high burden of disability-adjusted life years and premature mortality from non-communicable chronic diseases ^(57)^. Therefore, our findings highlight the need for additional multifaceted initiatives, including individual- and environmental-level interventions and policy changes, so that better quality dietary habits are adopted from childhood. In particular, these initiatives and policies need to target children and their parents from lower SEP. This is especially important considering the large burden of dietary-related chronic diseases in the UK, which contribute to social inequalities in health. Finally, the C-EWG score is useful tool which can be used in future research to explore how overall alignment to UK dietary guidelines during childhood relates to current and future health.

## Supplementary material

For supplementary material accompanying this paper visit https://doi.org/10.1017/S0007114522003336. click here to view supplementary material
