# Supplementary material for: Adherence to UK dietary guidelines in school-aged children from the Avon Longitudinal Study of Parents and Children (ALSPAC) cohort
Source: Br J Nutr. 2022 Oct 28;130(3):454–66. doi: 10.1017/S0007114522003336 (PMC10331434; doi:10.1017/S0007114522003336)

**Supplementary Material**

**Adherence to UK dietary guidelines in school-aged children from the Avon Longitudinal Study of Children and Parents (ALSPAC) cohort.**

Genevieve Buckland^1^, Kate Northstone^2^, Pauline M Emmett^1^, Caroline M Taylor^1^

**^1^Centre for Academic Child Health, Bristol Medical School, University of Bristol**

**^2^**Department of Population Health Sciences**, Bristol Medical School, University of Bristol**

**Supplementary Methods: Calculation of Child-Appropriate Portion Sizes: Fruit and vegetables, Fish, and Red and Processed Meat.**

The adult portion sizes for fruit and vegetables, fish, and red and processed meat outlined by UK Health Security Agency (formerly known as Public Health England) [1] were adjusted to child-appropriate portion sizes for the 7-, 10- and 13-year-old children in the ALSPAC cohort, based on a previously published method [2]. Firstly, the mean dietary reference value (DRV) for energy intake for 19–54-year-old men and women was obtained from the Scientific Advisory Committee on Nutrition (SACN) report on the DRVs for energy [3]. Secondly, using this DRV for adult energy intake (10.1MJ/day) and the recommended adult portion sizes [4], the relative portion size in grams per 1.0 MJ of intake was calculated for each food group (i.e. 140g fish divided by 10.1MJ/day=14g/MJ, 80g fruit or vegetables divided by 10.1MJ/day=8g/MJ and 70g red and processed meat divided by 10.1MJ=7g/MJ). Thirdly, in order to rescale the portions sizes to child-appropriate sizes their age-specific energy requirements were considered. The weight (g) per MJ for each food item or group (calculated above) was multiplied by the energy intake recommended for children at each age group (MJ) (mean energy DRV for boys and girls at 7-years-old = 6.7MJ/day, 10-years-old = 8.3MJ/day and 13-years-old = 9.7MJ/day). For example, 14g/MJ of fish was multiplied by 6.7MJ/day, equating to a portion of fish for 7-year-olds defined as 95g (rounded to nearest 5g). This resulted in portion sizes which were proportionally less at each age.

1. Levy L, Tedstone A (2017) UK Dietary Policy for the Prevention of Cardiovascular Disease. Healthcare (Basel) 5 (1). doi:10.3390/healthcare5010009

2. Jones LR, Steer CD, Rogers IS, Emmett PM (2010) Influences on child fruit and vegetable intake: sociodemographic, parental and child factors in a longitudinal cohort study. Public Health Nutr 13 (7):1122-1130. doi:10.1017/S1368980010000133

3. Public Health England. Scientific Advisory Committee on Nutrition: Dietary Reference Values for Energy. (2011). <https://www.gov.uk/government/publications/sacn-dietary-reference-values-for-energy>. Accessed 29.11.21

4. Public Health England. The Eatwell Guide: How does it differ to the eatwell plate and why? (2016). <https://assets.publishing.service.gov.uk/government/uploads/system/uploads/attachment_data/file/528201/Eatwell_guide_whats_changed_and_why.pdf>. Accessed 27.09.21

**Supplementary Table 1.** Comparison of baseline characteristics of ALSPAC children with incomplete and implausible dietary data and ALPSAC children with complete and plausible dietary data at 7 years (A) and at 13 years (B).

**A.**

**B.**

**Supplementary Table 2.** Alignment to UK dietary guidelines for total fat, saturated fat and free sugars, comparing sex- and age-specific grams per day limits with adherence using percentage of food energy limits, in the ALSPAC children at 7, 10 and 13 years.

**Supplementary Figure 1.** Proportion of ALSPAC children at 7-, 10- and 13-years with complete dietary data classified as being under-reporters, plausible reporters and over-reporters of total dietary intake.

**
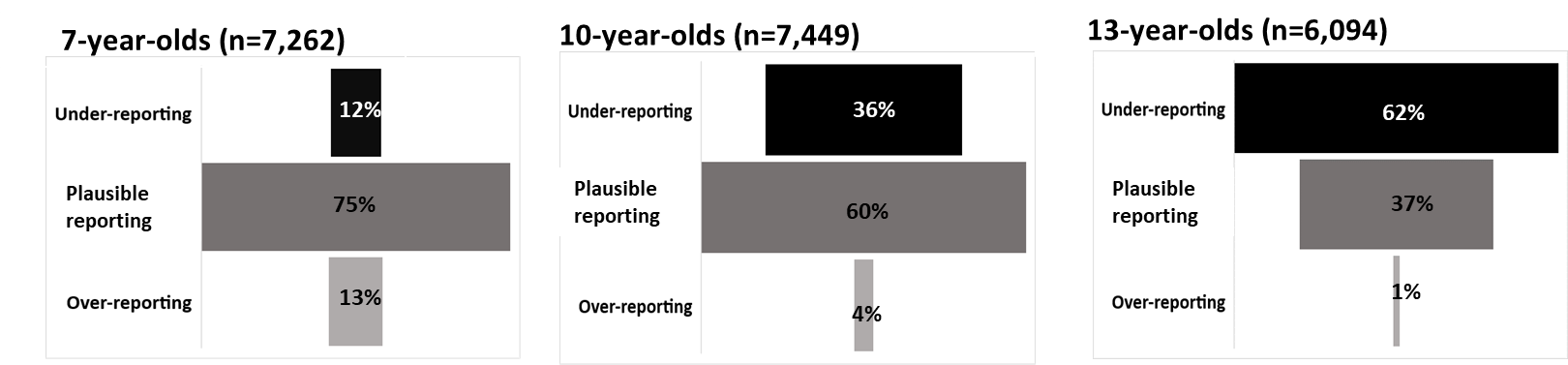
**

**Supplementary Figure 2.** Percentage of ALSPAC participants adhering to core Eatwell Guide UK dietary recommendations at 7 years (A), 10 years (B) and 13 years (C), comparing all participants (complete cohort) to children classified as plausible dietary reporters.

**A. B. C.**


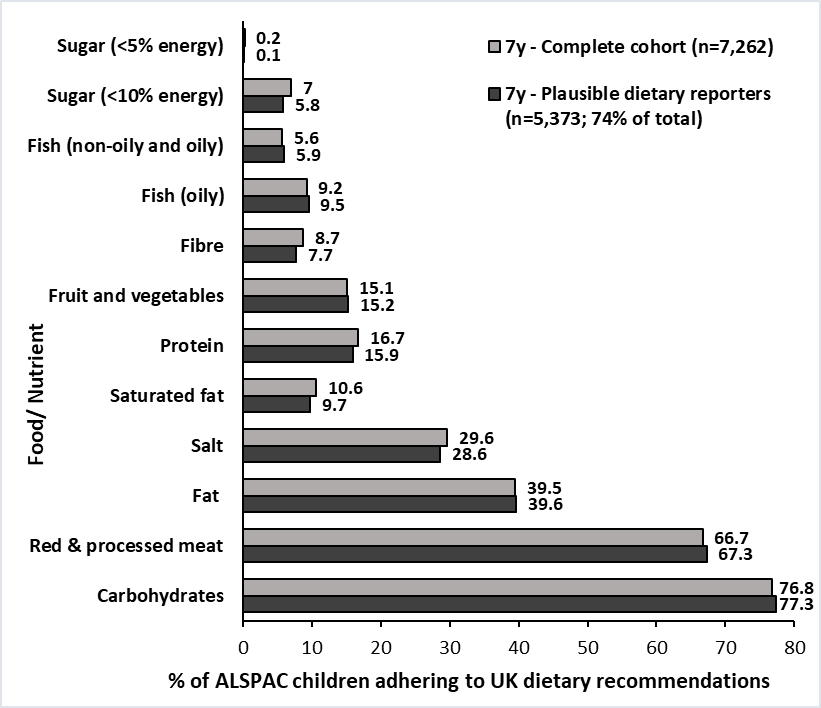

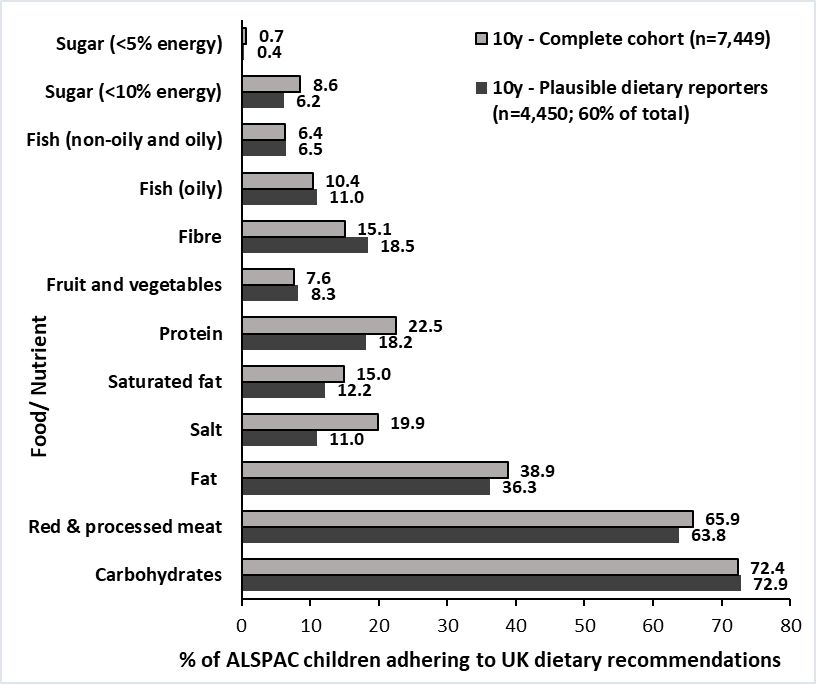

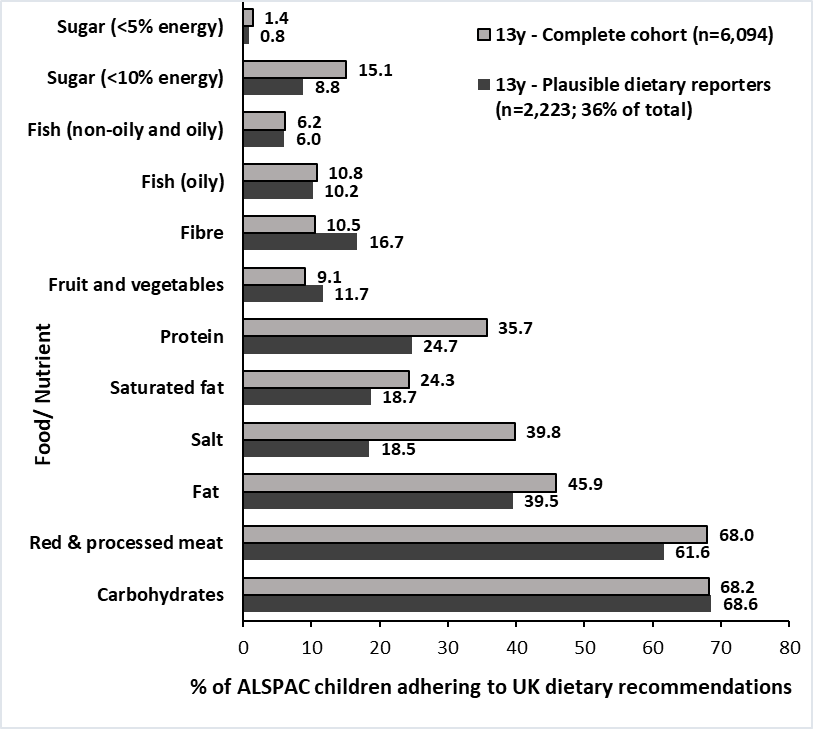

Supplement: Supplementary file 1 [file S0007114522003336sup.zip › S0007114522003336sup001.docx]
